# MITF depletion elevates expression levels of ERBB3 receptor and its cognate ligand NRG1-beta in melanoma

**DOI:** 10.18632/oncotarget.10422

**Published:** 2016-07-06

**Authors:** Tine N. Alver, Timothy J. Lavelle, Ane S. Longva, Geir F. Øy, Eivind Hovig, Sigurd L. Bøe

**Affiliations:** ^1^ Department of Tumor Biology, Institute for Cancer Research, Oslo University Hospital, Oslo, Norway; ^2^ Institute for Cancer Genetics and Informatics, The Norwegian Radium Hospital, Oslo University Hospital, Oslo, Norway; ^3^ Department of Informatics, University of Oslo, Oslo, Norway

**Keywords:** MITF, ERBB3, NRG1-beta, PI3K signaling, melanoma

## Abstract

The phosphatidylinositol-4,5-bisphosphate 3-kinase (PI3K) pathway is frequently hyper-activated upon vemurafenib treatment of melanoma. We have here investigated the relationship between SRY-box 10 (SOX10), forkhead box 3 (FOXD3) and microphthalmia-associated transcription factor (MITF) in the regulation of the receptor tyrosine-protein kinase ERBB3, and its cognate ligand neuregulin 1-beta (NRG1-beta). We found that both NRG1-beta and ERBB3 mRNA levels were elevated as a consequence of MITF depletion, induced by either vemurafenib or MITF small interfering RNA (siRNA) treatment. Elevation of ERBB3 receptor expression after MITF depletion caused increased activation of the PI3K pathway in the presence of NRG1-beta ligand. Together, our results suggest that MITF may play a role in the development of acquired drug resistance through hyper-activation of the PI3K pathway.

## INTRODUCTION

Malignant melanoma is an aggressive cancer form with limited treatment options and poor survival for patients with advanced disease. The small-molecular inhibitor vemurafenib, that specifically inhibits the BRAFV600 mutated protein function, has shown remarkable response when used in patients [1–3]. Unfortunately, virtually all responders acquire resistance over time [4]. Several resistance mechanisms have been characterized [5–11], including receptor tyrosine kinase (RTK) up-regulation or activation leading to sustained cell survival and proliferation [12, 13]. Two recent reports have suggested that microphthalmia-associated transcription factor (MITF) loss is involved in the development of vemurafenib resistance through up-regulation or activation of RTKs such as AXL and EGFR [12, 13].

The EGFR family members of RTKs consist of four family members; receptor tyrosine-protein kinase ERBB1 to 4 (ERBB1/EGFR, ERBB2/HER2, ERBB3/HER3, ERBB4/HER4, respectively). These receptors are important regulators of normal growth and cell differentiation. Their dysregulation in the form of amplification, overexpression or mutation is associated with tumor development and poor clinical prognosis in most human cancers [14]. Previous studies have suggested that increase in both EGFR and ERBB3 expression and phosphorylation can be induced after mitogen-activating protein kinase (MAPK) inhibitor treatment [12, 15–20], and that targeting EGFR and ERBB3 can prevent and extend the establishment of MAPK inhibitor-induced resistance in melanoma [12, 15, 17]. Transcription factors such as FOXO1, FOXO3A, FOXD3, and SOX10 have been reported to activate ERBB3 expression [15, 21–23]. Interestingly, SOX10 has been found to activate MITF expression [24], while MITF is reported to repress FOXD3 expression and *vice versa* [25, 26].

Preliminary gene expression arrays suggested to us that ERBB3 expression was elevated after MITF depletion in the SKMEL28 cell line. Based on these data, we have here investigated the relationship between SOX10, FOXD3 and MITF in the regulation of the receptor tyrosine-protein kinase ERBB3, and its cognate ligand NRG1-beta. We found that depletion of MITF protein resulted in elevation of ERBB3 and NRG1-beta levels. The novel mechanism described here may have implications for the development of acquired drug resistance in melanoma.

## RESULTS

### Basal expression levels of SOX10, MITF, FOXD3 and ERBB3 in a melanoma cell line panel

**Figure 1:**
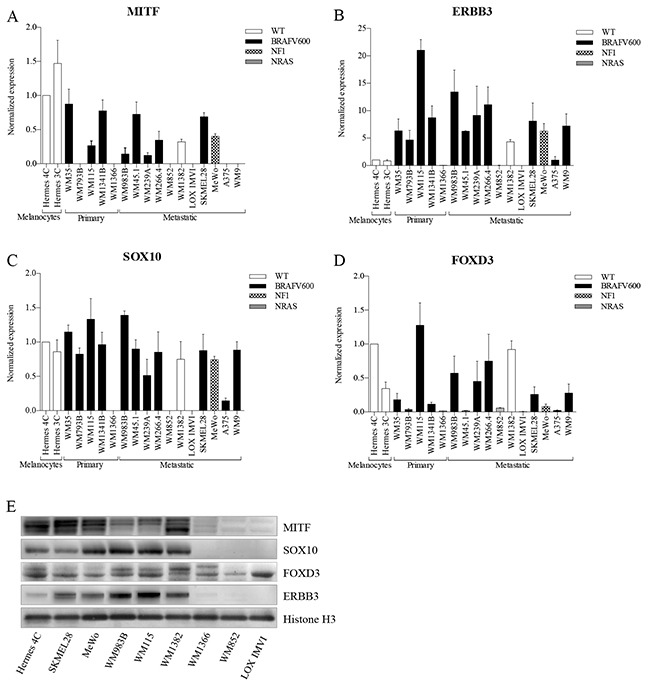
Basal expression levels of MITF, ERBB3, SOX10 and FOXD3 in various melanoma cell lines **A-D.** qRT-PCR was used to evaluate mRNA levels of MITF **(A)**, ERBB3 **(B)**, SOX10 **(C)**, and FOXD3 **(D)** in melanoma cell lines by normalizing against immortalized cultured melanocytes (Hermes 4C). Bars represent mean ± SD of three separate experiments **(E).** Representative western blots of MITF, SOX10, FOXD3 and ERBB3 protein levels shown in 9 different cell lines representing various disease stage and genetic background. Histone H3 was used as loading control.

We compared basal mRNA expression levels of SOX10, MITF, FOXD3, and ERBB3 in immortalized melanocytes, and in a panel of melanomas spanning various genetic backgrounds (see Figure 1A-1D and Supplementary Table S1). Out of the 18 cell lines analyzed for mRNA expression, we selected 9 cell lines for further protein expression analysis, representing various alterations in the MAPK pathway (NRAS, BRAF, NF1) and variable MITF expression levels (Figure 1E). We found that mRNA and protein expression levels correlated well for all cell lines tested. FOXD3 and SOX10 have been reported to be activators of ERBB3 transcription [15, 21], which is in agreement with what we observed, as depletion of FOXD3 and SOX10 levels resulted in reduced ERBB3 expression. To our knowledge, no reports exist concerning MITF regulation of ERBB3. Our results show that MITF protein expression has an inverse association with ERBB3 protein expression in the MITF-expressing cell lines, particular in the immortalized melanocyte cell line Hermes 4C, WM983B and WM115 (Figure 1E).

### Depletion of MITF elevates ERBB3 expression at the transcriptional level

**Figure 2:**
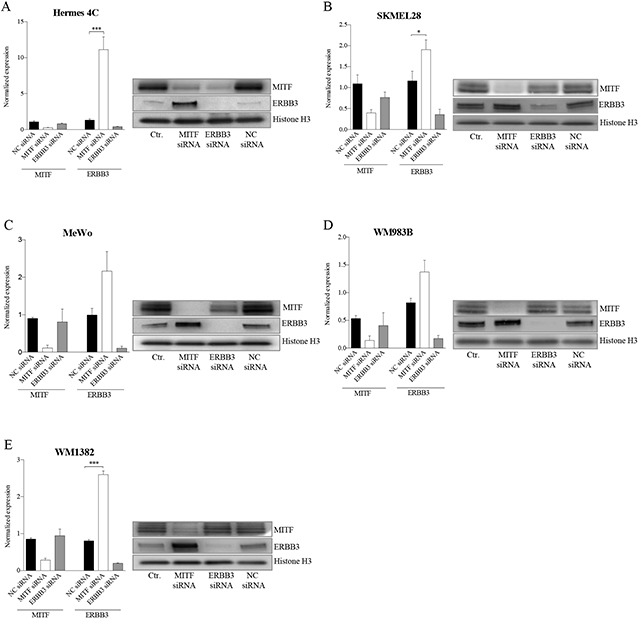
MITF suppresses ERBB3 expression at the transcriptional level in various cell lines after siRNA transfections Assessment of mRNA and protein levels of MITF and ERBB3 in a panel of cell lines 72h after siRNA-induced reduction of MITF and ERBB3. **A.** Hermes 4C (immortalized melanocytes). **B.** SKMEL28 (BRAFV600E) **C.** MeWo (NF1) **D.** WM983B (BRAFV600E) **E.** WM1382 (wild-type for BRAF and NRAS). Graphs represent qRT-PCR expression data from three separate experiments normalized to untreated control cells and plotted as mean ± SD. * =*p* < 0.05, *** =*p* <0.005 (*t*-test). A representative western blot of the corresponding treatments is shown to the right, with Histone H3 as loading control.

To further explore the relationship between MITF and ERBB3, we depleted MITF and ERBB3 levels by the use of siRNA molecules in five MITF-expressing cell lines differing in MAPK pathway backgrounds (see Supplementary Table S1). Elevation of the ERBB3 mRNA and protein levels were detected 72h post siMITF treatment for all five cell lines tested (Figure 2A-2E). Transfection of the same cell lines with siERBB3 resulted in reduction of MITF protein levels in Hermes 4C and MeWo, while no changes were observed in WM1382, WM983B and SKMEL28. To ensure that the elevated ERBB3 levels after siMITF treatment was not caused by an off-target effect, we also tested two other siMITF sequences and an additional negative siRNA control. All the three individual siMITF molecules resulted in elevation of ERBB3, compared to untreated control and negative siRNA controls (Supplementary Figure S1). In addition to siMITF treatment, we also overexpressed the melanocyte-specific variant 4 (NM_000248) MITF protein by MITF mRNA delivery, resulting in reduction of ERBB3 mRNA levels after 24h in A375 and MeWo (See Supplementary Figure S2).

### Elevation of the ERBB3 receptor expression levels increases pAKT levels

**Figure 3:**
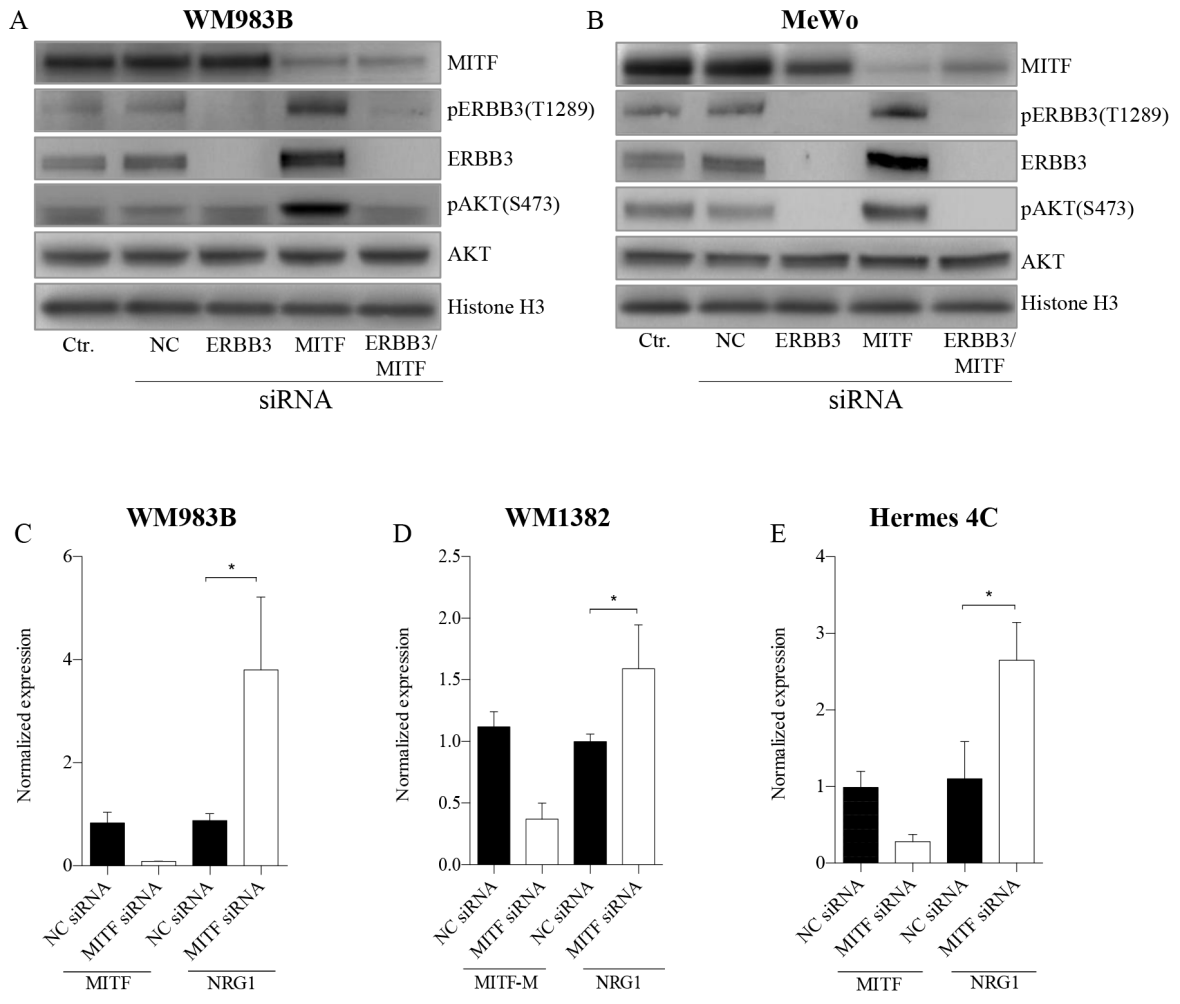
MITF suppress the PI3K-pathway through NRG1-beta/ERBB3 signaling **A-B.** Representative western blots show the effect of MITF siRNA treatment on NRG1-beta/ERBB3 signaling pathway members, leading to p-AKT (S473) activation in WM983B **(A)** and MeWo **(B)**. Cell lines were transfected with MITF and ERBB3 siRNA alone and in combination for 72h, and treated either with or without (see Supplementary Figure S3A-S3B) 10ng/ml NRG1-beta ligand 15min prior to harvesting. All experiments were performed in triplicate. Histone H3 was used as loading control. **C-D.** qRT-PCR data show mRNA elevation of NRG1-beta ligand after MITF depletion in WM983B **(C)**, WM1382 **(D)** and Hermes 4C **(E).** Graphs represent qRT-PCR expression data from three separate experiments normalized to untreated control cells and plotted as mean ± SD. * =*p* < 0.05 (*t*-test).

To address whether depletion of MITF protein activates the PI3K pathway through NRG1-beta/ERBB3 signaling in both mutant and wild-type BRAF melanoma cell lines, we co-transfected WM983B and MeWo with MITF and ERBB3 siRNA molecules in combination with NRG1-beta treatment (Figure 3A–3B), or without NRG1-beta treatment (See Supplementary Figure S3A-S3B). After 72h of siMITF treatment, we observed an induction of both phospho-ERBB3-T1289 (pERBB3) and total ERBB3 expression, together with an increase in pAKT (S473) in both cell lines tested. In contrast to siMITF transfection alone, co-transfection of the WM983B and MeWo cell lines with siMITF and siERBB3 resulted in loss of pERBB3 (T1289), total ERBB3 and pAKT (S473). In MeWo, a complete depletion of pAKT (S473) levels was observed after co-transfection, while only the hyper-activated levels of pAKT (S473) were depleted in the WM983B cell line. To address the observed increase in pERBB3 (T1289) levels after siMITF treatment observed in non-NRG1-beta treated WM983B samples (See Supplementary Figure S3A), we measured the NRG1-beta mRNA levels. We found an increase in NRG1-beta mRNA expression levels in WM983B (Figure 3C). No measureable levels of NRG1-beta mRNA were detected in MeWo by qPCR analysis. Since we did not detect any NRG1-beta in the BRAF wild-type cell line MeWo, we investigated two other BRAF wild-type cell lines, the metastatic melanoma cell line WM1382 (Figure 3D) and the immortalized melanocyte cell line Hermes 4C (Figure 3E). Also these cell lines showed elevation of NRG1-beta mRNA levels after MITF depletion. When overexpressing MITF protein by mRNA transfection in A375 harboring low MITF levels, we observed a minor reduction of NRG1-beta mRNA levels after 24h (See Supplementary Figure S2A).

### MITF regulates ERBB3 in a FOXD3 dependent and independent manner

**Figure 4:**
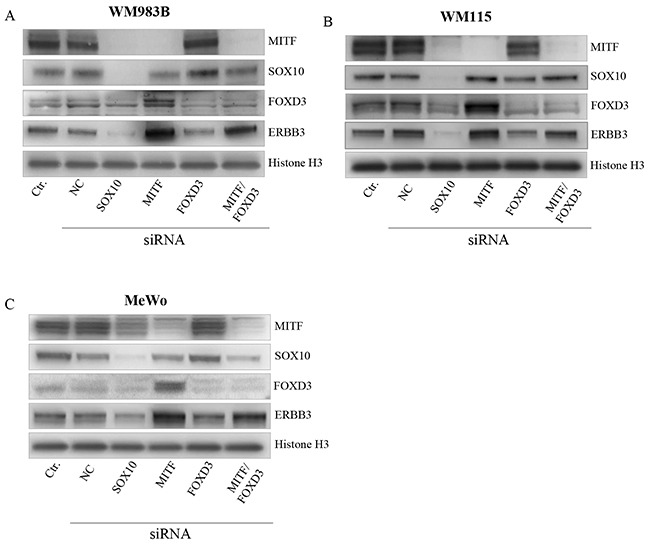
MITF suppress ERBB3 expression, both through and without FOXD3 Representative western blot showing the effect of SOX10, MITF and FOXD3 siRNA treatment upon ERBB3 expression after 72h in WM983B **(A).** WM115 **(B).** and MeWo **(C).** siRNA treatment was performed in triplicate. Histone H3 was used as loading control.

To investigate the role of SOX10, MITF, and FOXD3 and the reported effects due to BRAF status in ERBB3 regulation, we depleted the three former genes individually and in combination for MITF/FOXD3 by the use of siRNA in three melanoma cell lines: WM983B, MeWo and WM115. MeWo represent BRAF wild type, while WM983B and WM115 harbor mutant BRAF. Additionally, the WM115 cell line was utilized in a previous study by Abel et al. [15], were the authors reported that FOXD3 is an activator of ERBB3 expression only in V600 cell lines. Our results show that siSOX10 treatment reduces the MITF, FOXD3 and ERBB3 protein levels in all cell lines (Figure 4A–4C). In contrast, siMITF treatment resulted in an increase in both ERBB3 and FOXD3 protein levels (Figure 4A–4C). When transfecting the cell lines with siFOXD3 alone, no differences in ERBB3 protein levels were detected in MeWo compared to a negative siRNA control. However, we observed a minor reduction of ERBB3 protein levels in WM983B and WM115. To explore whether MITF depletion results in elevation of ERBB3 through FOXD3, we performed an siRNA co-transfection of MITF and FOXD3 in all three cell lines, demonstrating an increase in ERBB3 protein levels compared to negative siRNA control in WM983B and MeWo, while no change was detected in WM115.

### MITF binding sites in ERBB3, NRG1-beta and FOXD3

**Figure 5:**
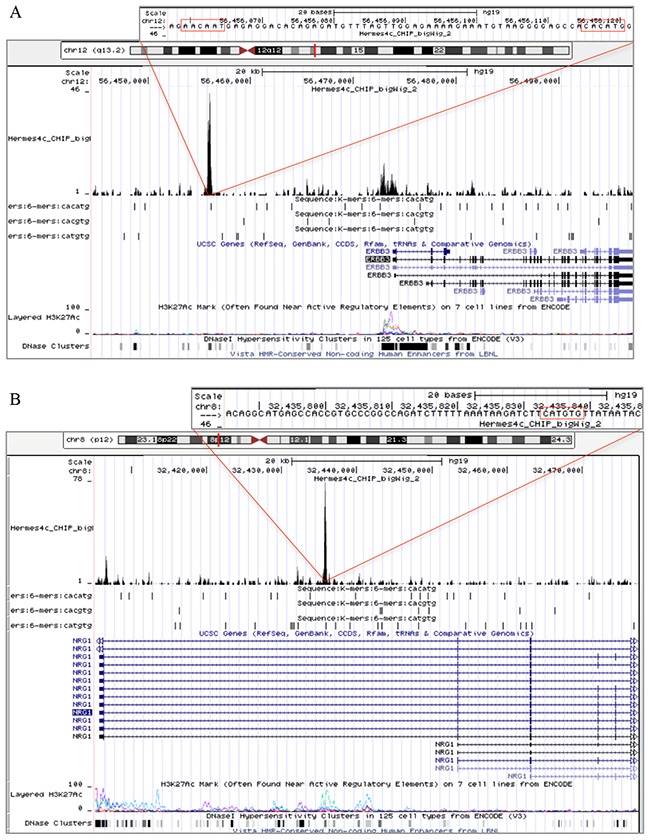
MITF binding sites in ERBB3, NRG1-beta and FOXD3 Snapshots from the University of California Santa Cruz (UCSC) genome browser of the ERBB3 **(A).** NRG1-beta **(B).** and FOXD3 MITF binding sites in ERBB3, NRG1-beta and FOXD3. **(C).** genes in Hermes 4c cells expressing 5′ HA-tagged MITF in the hg19 assembly. The figure includes tracks for MITF ChIP-seq, RefSeq Genes, three E-box motifs (CACATG, CACGTG and CATGTG), and ENCODE regulation (H3K27Ac/DNase clusters). The scale in the MITF ChIP-seq tracks indicates the intensity of MITF enrichment. (A): MITF ChIP-seq results shows that a MITF enriched site was found in the regulatory region of ERBB3, approximately 18kb upstream of the transcription start site of the gene. The MITF enriched site was co-localized with an E-box (CACATG) and the DNA binding motif of SOX10 (AACAAT). Enrichment of MITF was also detected in the promoter region of the ERBB3 gene. (B): A MITF enriched site together with an E-box (CATGTG) motif was found in the first intron of the NRG1-beta gene. (C): MITF enrichment together with an E-box (CACGTG) motif was found in the promoter region of FOXD3.

To address whether MITF protein binds DNA in the proximity of ERBB3, NRG1-beta, and FOXD3, we performed a 3xHA-tagged MITF ChIP-seq analysis in the Hermes 4C cell line. Results showed a strong MITF binding signal in the putative regulatory region, approximately 18kb upstream of the transcription start site, and two minor binding signals in the promoter region of ERBB3 (Figure 5A). In the putative regulatory region, we detected both an E-box (CACATG) and a SOX10 binding motif (AACAAT), while no E-boxes were found in the promoter region. In the NRG1-beta gene, we detected a strong MITF binding signal in the regulatory region within the first intron. This MITF binding signal contained two E-box motifs and was located approximately 30kb downstream of the transcription start site. Furthermore, we detected a minor MITF binding signal in the proximal promoter region of NRG1-beta, without any E-box motifs (Figure 5B). When investigating the FOXD3 gene, we found a MITF binding signal containing an E-box motif in the promoter region (Figure 5C).

### Correlation between SOX10 and ERBB3 in patient samples

**Figure 6:**
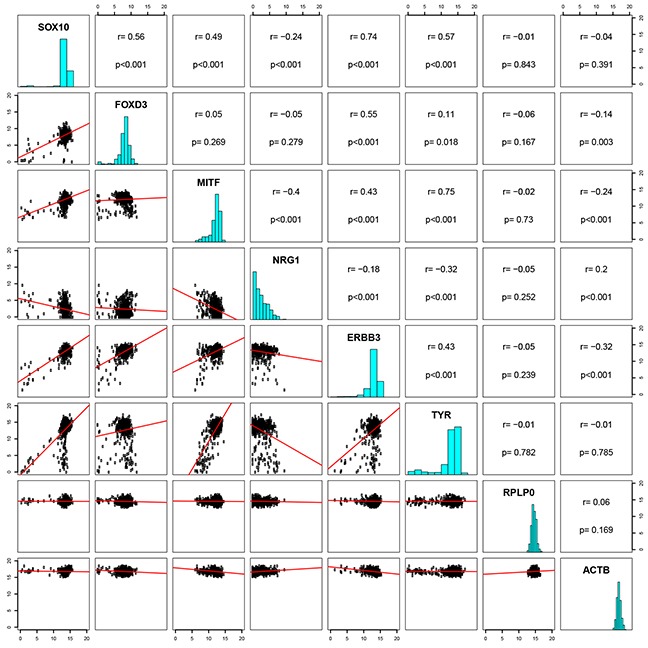
Correlation between SOX10 and ERBB3 in patient samples A pairwise correlation plot of the cutaneous skin cancer (n=473) exon expression cases of TCGA (data set available at https://tcga-data.nci.nih.gov/docs/publications/skcm_2015/). IlluminaHiSeq-defined RNAseq is shown for the genes SOX10, FOXD3, MITF, NRG1, ERBB3 and TYR are shown, in addition to two genes uncorrelated, RPLP0 and ACTB. Both axes display log2 expression values.

To investigate the relationship between SOX10, MITF, FOXD3, ERBB3 and NRG1 in patient samples, we utilized The Cancer Genome Atlas (TCGA, SKCM), which contains a set of 474 melanoma patient samples. A strong correlation (R = 0.74, P = <0.0001) was found between SOX10 and ERBB3 (Figure 6). Correlations were also detected between FOXD3 and ERBB3 (R = 0.55, P = <0.0001), SOX10 and FOXD3 (R = 0.56, P = <0.0001), and SOX10 and MITF (R = 0.49, P = <0.0001). Among the genes investigated, MITF and NRG1 showed weakest correlation with ERBB3, with R = 0.43 and R = 0.18, respectively. Interestingly, we observed an inverse correlation between MITF and NRG1 (R = −0.4, P = <0.0001) in the TCGA data. TYR, in addition to two genes assumed uncorrelated to MITF, RPLPO and ACTB, were included as controls.

### Vemurafenib treatment results in loss of MITF and elevation of FOXD3 and ERBB3

**Figure 7:**
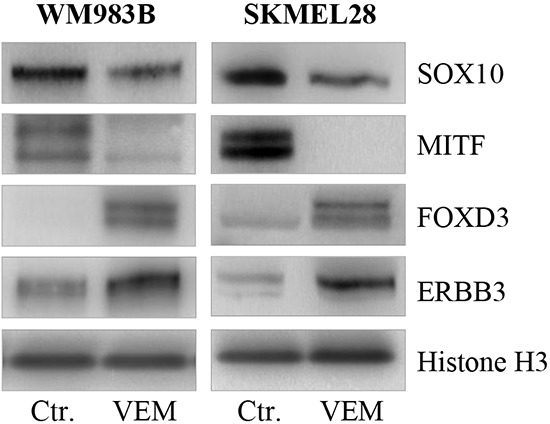
Vemurafenib treatment results in loss of MITF and gain of FOXD3 and ERBB3 Representative western blots showing MITF loss and subsequent gain of ERBB3 and FOXD3 protein after 2 weeks of vemurafenib treatment (0.5μM) in the WM983B and SKMEL28 cell lines. Vemurafenib-cultured cells were performed in triplicate. Histone H3 was used as loading control.

As vemurafenib has been reported to induce loss of MITF, we measured the protein expression levels of MITF, SOX10, FOXD3 and ERBB3 after inhibitor treatment in the WM983B and SKMEL28 cell lines (Figure 7). An extensive decrease in MITF protein levels after two weeks of vemurafenib treatment was observed in both cell lines, while a minor reduction in SOX10 levels could be detected. In contrast, we measured elevated ERBB3 and FOXD3 protein levels in the same samples.

## DISCUSSION

In this study, we have demonstrated that the ERBB3 receptor and its cognate ligand NRG1-beta are elevated after MITF depletion in various melanoma cell lines. Our data is supported by previous studies showing that both ERBB3 expression and phosphorylation can be elevated after MAPK inhibitor treatment [15–20]. Hyper-activation of the PI3K/AKT pathway due to increased NRG1-beta/ERBB3 pathway signaling has previously been reported to be responsible for acquired drug resistance in BRAFV600 melanomas [15, 17]. However, also low MITF/AXL and MITF/EGFR ratios have been suggested to be involved in early resistance to vemurafenib in a subset of melanomas [12, 13]. Together, these reports suggest that MITF loss after vemurafenib treatment, followed by PI3K/AKT elevation, is a key element to overcome drug treatment and in acquiring drug resistance against MAPK inhibitors. Both gain and loss of MITF after vemurafenib has been reported [13]. The molecular mechanism behind these alterations in MITF levels is presently not known. In the SKMEL28 and WM983B cell lines, we observed that SOX10, an activator of MITF transcription, was reduced after vemurafenib treatment. However, as also both gain and loss of SOX10 protein after vemurafenib-induced MITF loss have been reported in the literature [13], the role of SOX10 in vemurafenib-induced MITF loss remains unclear.

SOX10 and FOXD3 have been reported to activate ERBB3 expression [15, 21], in addition to also activating and repressing MITF expression, respectively [24, 25, 27]. In this context, we investigated their role in ERBB3 regulation. When depleting FOXD3 in the two BRAFV600 cell lines WM983B and WM115, we detected a minor reduction of ERBB3 protein, which is in agreement with what Abel *et al*. [15] measured in the WM115 cell line. In the MeWo cell line, FOXD3 depletion alone did not affect ERBB3 protein expression. This is supported by Abel and Aplin [28], who showed that FOXD3 does not regulate ERBB3 expression in BRAF wild-type melanoma cells, nor in melanocytes. Furthermore, our siRNA depletion results demonstrated that SOX10 and MITF have opposite roles in the regulation of FOXD3 and ERBB3 expression. The observed FOXD3 elevation after MITF depletion is in agreement with He *et al*. [26] and implies that MITF can repress ERBB3 expression indirectly by suppressing FOXD3 in V600 cell lines. However, our data also demonstrate that MITF protein can bind directly to the regulatory region of ERBB3, as well as NRG1-beta and FOXD3. Together with our siRNA experiments, these latter results suggest that MITF can repress ERBB3 both with and without FOXD3 involvement, depending on the BRAF mutational status, as shown in this study. In the regulatory region of ERBB3, we detected a SOX10 binding site 40 bp downstream of the MITF-binding E-box, suggesting SOX10/MITF co-localization. Co-localization of MITF and SOX10 in the form of MITF-associated regulatory elements (MARE), where MITF alone, or with SOX10, binds between two BRG1-bound nucleosomes, has previously been described [29]. However, the precise mechanism(s) by which MITF represses ERBB3, FOXD3 and NRG1-beta expression remains the subject of ongoing investigation. An inverse relationship between MITF and ERBB3 protein expression levels was observed in our cell panel, and this was particularly evident in the immortalized melanocyte cell line Hermes 4C, and in the melanoma cell lines WM983B and WM115. Simultaneously, our results show that high MITF protein levels, in Hermes 4C, are not able to completely abolish ERBB3 transcription, implying the existence of a threshold with respect to MITF-induced repression of ERBB3 expression. This is also supported by the moderate suppression of ERBB3 expression detected when overexpressing MITF protein in MeWo and A375. We also observed an inverse correlation between MITF and NRG1-beta in the 474 patient samples from TCGA. In contrast, no inverse correlation was observed between MITF and ERBB3 in the TCGA samples. The reason for the lack of inverse correlation between MITF and ERBB3 in patient samples is not known, but could result from the fact that MITF suppression of ERBB3 seems to be only partial, and to depend on SOX10 expression. SOX10-dependent MITF repression of ERBB3 is supported by the observation that cell lines without both SOX10 and MITF (WM1366, WM852, LOXIMVI) lack ERBB3.

In summary, we have in this study shown that MITF depletion results in elevated expression levels of both the ERBB3 receptor and its cognate ligand NRG1-beta, which may have potential implications for acquired drug resistance in melanoma.

## MATERIALS AND METHODS

### Cell lines and culture conditions

The SKMEL28, MeWo, and A375 cell lines were obtained from the American Type Culture Collection (ATCC; Rockville, MD, USA), WM35, WM115, WM1341B, WM1366, WM983B, WM45.1, WM239A, WM266.4, WM852, WM1382, WM9, WM793B from the Wistar Institute, and LOXIMVI established in-house. For genetic background information, see Supplementary Table S1. The immortalized melanocyte cell line Hermes 3C and 4C were purchased from Wellcome Trust Functional Genomics Cell Bank [30]. Hermes 3C and 4C were cultured and maintained in 254 medium (Gibco, Invitrogen, Oslo, Norway) supplemented with 10% fetal calf serum (FCS) (PAA, New Bedford, MA, USA), 200nM phorbol-12-myristate-13-acetate (TPA; Sigma-Aldrich), 200pM (Hermes 3C) and 2pM (Hermes 4C) cholera toxin (Sigma-Aldrich), 10ng/ml human stem cell factor (hSCF; Thermo Fisher) and 10nM endothilin (EDN1; Millipore, Oslo, Norway) with the exception of where noted in the text. All melanoma cell lines were cultured and maintained in RPMI 1640 medium (Bio Whittaker, Verviers, Belgium), supplemented with 10% fetal calf serum (FCS) (PAA, New Bedford, MA, USA) and 2mM GlutaMAX (Gibco, Invitrogen, Oslo, Norway) with the exception of where noted in the text. The cells were maintained at 37°C in a humidified atmosphere containing 5% CO_2_ and were routinely tested for *Mycoplasma* infections (VenorGeM, Minerva Biolabs, Berlin, Germany), and the cell line identities were also verified by short tandem repeat (STR) analysis.

### Transfection and RNA interference

Cells were seeded in 6-well plates and grown to 60% confluence before being transfected and incubated for 72h with siRNA directed against MITF-M and SOX10 (Eurogentec, Seraing, Belgium), ERBB3 (Life Technologies) and FOXD3 (Dharmacon). The reason for choosing 72h incubation time was to ensure optimal reduction of our targets at the protein level. The cells were transfected with a final concentration of 25pmol siRNA, using Lipofectamine RNAiMAX (Invitrogen) as described in the manufacturers protocol. Sequences for siRNA can be found in the Supplementary Methods. All siRNA transfections were performed in triplicate.

### Western immunoblotting

Preparations of whole cell lysates were performed by addition of lysis buffer (150mM NaCl, 50mM Tris-HCl pH 7.5, 0.1% Nondient P-40, 1x Complete tablets mini EASYpack and 1x PhosSTOP from Roche), to frozen, dry cell pellets. The samples were left on ice for 30min with intermittent vortexing, followed by sonication and centrifugation to remove cell debris. Protein quantification was done using a standard Bradford protein assay (Bio-Rad Laboratories, Inc., CA). Protein lysates (30μg) were separated on 4-12% NuPAGE® Novex Bis-Tris Midi-Gels (Invitrogen, Carlsbad, CA) and then transferred to a membrane using an iBlot Dry Blotting system (Invitrogen, Carlsbad, CA) according to the manufacturers instruction. The membranes were subsequently blocked in TBS/T (137mM NaCl, 20mM Tris-HCl pH 7.5 and 0.1% tween20) containing 5% BSA (Sigma-Aldrich, Steinheim, Germany) for 1h, and then incubated with primary antibodies over night at 4°C with gentle agitation. Residual primary antibodies were removed by washing 3 × 10min in TBS/T. The membranes were thereafter incubated for 1h at RT with horse radish peroxidase conjugated (HRP) secondary antibody. The membranes were then washed 3 × 10min with TBS/T. The protein bands were visualized in the G-Box iChemi Chemiluminescence Image Capture (Syngene, England) using the SuperSignal chemiluminescence detection system (Pierce).

### Antibodies and inhibitors

The following antibodies were purchased from Cell Signaling Technology (Danvers, MA, USA); MITF (1:1000; #12590), Her3/ERBB3 XP (1:1000; #12708), Phospho-Her3/ERBB3-Tyrosine-1289 (1:1000; #4791), FOXD3 (1:1000; #2019), SOX10 (1:1000; #14374), Akt (1:2000; #9272), Phospho-Akt-Serine-473 XP (1:2000; #4060), and Histone H3 (1:3000; #4499) was used as loading control. Secondary antibody against rabbit (1:5000; P0448) was purchased from Dako (Agilent Technologies, Glostrup, Denmark). The small molecular inhibitor vemurafenib (Plexxikon 4032) was obtained from Selleck Chemicals (Houston, TX, USA). The NRG1-beta ligand was purchased from ImmunoTools GmbH (Friesoythe, Germany). The NRG1-beta ligand was used to enhance the pERBB3 (T1289) signal, as our Phospho-Her3/ERBB3-Tyrosine-1289 antibody did not detect pERBB3 (T1289) levels without NRG1-beta addition (see also Supplementary Figure S3A-S3B). The reason for choosing 15min of incubation time and a NRG1-beta ligand concentration of 10ng/ml was due to a previous study where they showed efficient activation of the ERBB3 receptor in the WM115 cell line under these conditions [18].

### Quantitative reverse transcriptase PCR

Total cellular RNA was isolated with the GenElute Mammalian Total RNA Miniprep Kit (Sigma-Aldrich, Steinheim, Germany) and the qScript™ cDNA Synthesis Kit (Quanta Biosciences, Gaithersburg, USA) was used for reverse transcription. Both kits were used according to the manufacturers manuals. RNA concentration was measured using NANODROP 2000 (Thermo Scientific). Real-time detection was obtained by use of SYBR Green. For each PCR, 100ng cDNA, 30μl PerfeCTa™ SYBR® Green SuperMix for iQ (Quanta Biosciences, Gaithersburg, USA), 300nM of each primer and nuclease-free water was added to a final volume of 60μl. The final volume of 60μl was then split into two parallels of 25μl and added to the PCR plate. Primers against MITF-M, ERBB3, SOX10, FOXD3 and NRG1-beta were ordered from Integrated DNA technologies (IDT). Real-time reactions were run on a CFX Connect Real-Time PCR Detection System (Bio-Rad) with the following amplification protocol: 3 min initial denaturation at 95°C, 40 cycles of 15s denaturation at 95°C and 35s annealing/extension at 60°C, one hold at 95°C for 10 s followed by a hold for 30s at 60°C. Finally, a melt curve analysis was performed, starting at 60°C, increasing with 0.5°C steps (8s) until reaching a final temperature of 95°C. The quality of the RNA samples was verified by amplification of two housekeeping genes, the TATA-binding protein (TBP) and the human acidic ribosomal phosphoprotein PO (RPLPO). These housekeeping genes were chosen as they were unchanged by the different treatment modalities in this study (data not shown). Bio-Rad, CFX manager 3.1, was used for the quantitative calculations. The program performs calculations based on the ΔΔ C_T_ method [31], which allows comparison of cycle threshold values obtained using different sets of primers on the same set of samples.

### Chromatin immuno-precipitation sequencing (ChIPseq) protocol

Hermes 4C was transduced by lentiparticles carrying pLX3xHAvar4mCherry. Stable cell lines expressing the 3xHA tagged MITFvar4 were subsequently sorted based on the presence of mCherry and used further (sorted by Flow Cytometry Core Facility, OUS, Norway). Chromatin immuno-precipitation sequencing (ChIPseq) experiments were performed twice as described by Dahl JA and Collas P [32] with modifications. Briefly, chromatin from native Hermes 4C and Hermes 4C cells (mycoplasma unverified) stably transfected with a lentiviral vector expressing 3xHA-tagged MITF were fixed with 1% formaldehyde for 8min to cross-link DNA and proteins. Cross-linking was stopped with 125mM glycine for 5min. Cells were rinsed twice in phosphate buffered saline (PBS), harvested using trypsination and sedimented. Cells were resuspended in ice-cold lysis buffer (1% SDS, 10mM EDTA, 50mM Tris–HCl, pH 8.0) containing protease and phosphatase inhibitors. Cells were sonicated at 4°C using a Covaris S2 instrument to yield chromatin fragments of 300–500 bp. Chromatin was cleared by centrifugation, concentration determined by A260, and chromatin was diluted in RIPA buffer (0.1% SDS, 0.1% Na-deoxycholate, 1% Triton X-100, 1mM EDTA, 0.5mM EGTA, 140mM NaCl, 10mM Tris–HCl, pH 8.0) to 2 A260 units. Each immunoprecipitation was performed in 250μl RIPA buffer with 1μg antibody overnight at 4°C. The antibody: anti-HA-tag (12CA5, Roche) was used. Antibody-bound chromatin was precipitated using Dynabeads protein G (Invitrogen) and washed 3 times in RIPA buffer, once in TE buffer (10mM Tris–HCl, pH 8.0, 10mM EDTA) and eluted in 1% SDS with 100mM NaHCO3. Eluted chromatin was incubated at 65°C with proteinase K (Sigma-Aldrich; 50μg/ml) over night, and DNA was purified by phenol-chloroform-isoamylalcohol extraction (25:24:1) and once with chloroform/isoamylalcohol (24:1) followed by ethanol precipitation. DNA was dissolved in H2O and used for high-throughput sequencing using an Illumina Hiseq 2500 sequencer. The sequencing reads were aligned to hg19 using Bowtie v.1.0.0, after which MACS v 1.4.2 was used to call peaks. To get a common set of peaks for the two samples, the two. bed files containing the peaks were put together and sorted before using BEDTools v2.7.1 to merge them. IntersectBed was then used to get the number of reads falling into each peak for all samples. The counts were read into R, where two sets of normalized counts were computed, one using size normalization and one using quantile normalization from the package preprocessCore. Logfold2 change was computed for the two CHIPed samples. CHIP vs INPUT counts were plotted for Hermes4c and histograms were made of the log fold change values.

### Construction of pLX3xHAvar4mCh

The construction of pLX 3xHAvar4mCh was performed by two sequential clonings. The human MITF variant 4 cDNA was prepared by PCR amplification of the cDNA clone MGC:75121 (IMAGE:6066096) (Invitrogen) using the BglII containing MITF forward primer:5′-AAAAAAAGATCTACCATGCTGGAAATGCTAG-3′ and the EcoRI containing reverse primer: 5′-AAAAAGATTTCATCTCGCTAACAAGTGTGC. Subsequent to restriction digestion by BglII and EcoRI the insert was gel purified. The mouse equivalent of the human MITF var4 cDNA was removed from pCMV3xHAvar4 plasmid by BglII/ EcoRI endonuclease digestion [33]. The insert and vector were then ligated overnight at 14°C, transformed into chemically competent DH5α and selected candidates were screened by PCR for the presence of the insert. The successful construct pCMV3xHAvar4 was digested with NheI/XbaI and the 5′ 3xHA tagged MITF variant 4 fusion insert was isolated by gel purification. The insert was then ligated into gene cleaned (Genomed) SpeI linearized pLVX IRES mCh (Clontech) producing the Lenti-expression vector pLX3xHAvar4mCherry and transformed into chemically competent STBL3 (Invitrogen). pLX3xHAvar4mCherry was subsequently sequenced to confirm the correctness of the insertion.

### Production of lentiviral particles

Lentiparticles were produced in Lenti-X 293T (Clontech) using the designated lenti-expression vector and the second generation packaging system utilizing pCMV-dR8.2 dvpr and pCMV-VSV-G as previously described [34] with the following modification: The transfection reagent Fugene (Promega) was replaced by Turbofect (Thermo Fisher Scientific) and applied as recommended by the manufacturer.

### Statistical analysis

*P* values were calculated using a student t-test. Values *p* < 0.05 were considered statistically significant.

## SUPPLEMENTARY METHODS


