# Supplementary material for: MITF depletion elevates expression levels of ERBB3 receptor and its cognate ligand NRG1-beta in melanoma
Source: Oncotarget. 2016 Jul 6;7(34):55128–40. doi: 10.18632/oncotarget.10422 (PMC5342406; doi:10.18632/oncotarget.10422)
Supplement: Supplementary file 1 [file oncotarget-07-55128-s001.pdf]

MITF depletion elevates expression levels of ERBB3 receptor and its cognate ligand NRG1-beta in melanoma

SUPPLEMENTARY METHODS

RNA interference

MITF-M3 siRNA molecule sequence: Sense (5′- GCA-GUA-CCU-UUC-UAC-CAC-U -3′) anti sense (5′- AGU-GGU-AGA-AAG-GUA-CUG-C- 3′)

MITF-M1 siRNA molecule sequence: Sense (5′- GGU-GAA-UCG-GAU-CAU-CAA-G -3′) anti sense (5′- CUU-GAU-GAU-CCG-AUU-CAC-C- 3′)

MITF-M2 siRNA molecule sequence: Sense (5′- AGC-AGU-ACC-UUU-CUA-CCA-C -3′) anti sense (5′- GUG-GUA-GAA-AGG-UAC-UGC-U- 3′)

ERBB3 siRNA molecule sequence: Sense (5′- UCG-UCA-UGU-UGA-ACU-AUA-A- 3′) anti sense (5′- UUA-UAG-UUC-AAC-AUG-ACG-A -3′)

SOX10 siRNA molecule sequence: Sense (5′- GGU-CAA-GAA-GGA-ACA-GCA-G -3′) anti sense (5′- CUG-CUG-UUC-CUU-CUU-GAC-C -3′)

FOXD3 siRNA molecule sequence: M-009152-03-0010, siGenome Human FOXD3 (27022) siRNA-SMARTpool.

Quantitative reverse transcriptase PCR primers

Primers against MITF-M forward (5′-CAT-TGT-TAT-GCT-GGA-AAT-GCT-AGA-3′) and reverse (5′-GC-TAA-AGT-GGT-AGA-AAG-GTA-CTG-C-3′), MITF forward (5′-TTT-TCC-CAC-AGA-GTC-TGA -AGC-3′) and reverse (5′-TGT-TAA-ATC-TTC-TTC-TTC-GTT-CAA-TC-3′), ERBB3 forward (5′-CTG-ATC-ACC-GGC-CTC-AAT-3′) and reverse (5′-GGA-AGA-CAT-TGA-GCT-TCT-CTG-G-3′), SOX10 forward (5′-GAC-CAG-TAC-CCG-CAC-CTG-3′) and reverse (5′-CGC-TTG-TCA-CTT-TCG-TTC-AG-3′), FOXD3 forward (5′-GAA-GCC-GCC-TTA-CTC-GTA-CA-3′) and reverse (5′-CGC-TCA-GGG-TCA-GCT-TCT-T-3′), NRG1 forward (5′-CCC-ATG-AAA-GTC-CAA-AAC-CA-3′) and reverse (5′-CCG-GTT-ATG-GTC-AGC-ACT-CT-3′) TATA-binding protein (TBP) with forward primer 5′-GCC-CGA-AAC-GCC-GAA-TAT-3′ and reverse primer (5′-CGT-GGC-TCT-CTT-ATC-CTC-ATG-A-3′) and the human acidic ribosomal phosphoprotein PO (RPLPO) with forward primer (5′-CGC-TGC-TGA-ACA-TGC-TCA-AC-3′) and reverse primer (5′-TCG-AAC-ACC-TGC-TGG-ATG-AC-3′)

Production of mRNA molecules

3′3xHA-tagged MITFvar4 or 3′3xHA-tagged MITFdnR215del (dominant negative) mRNA molecules were produced as described previously [35].

mRNA transfection

Cells were seeded in 6-well plates and grown to 60% confluence before being transfected and incubated for 24h with Lipofectamine 2000 (Invitrogen) combined with 3′3xHA-tagged MITFvar4 or 3′3xHA-tagged MITFdnR215del (dominant negative) mRNA molecules according to the manufactures instructions.

SUPPLEMENTARY FIGURES AND TABLE

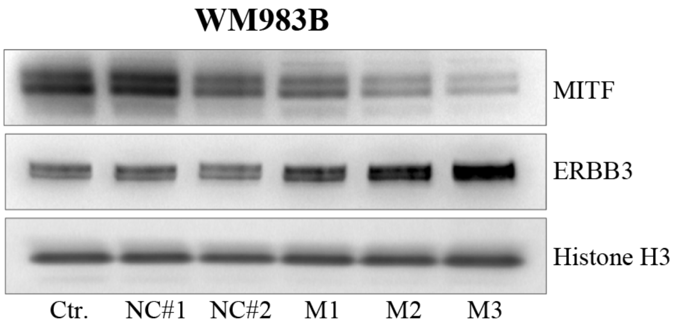

Supplementary Figure S1: Western blots showing the effect of negative siRNA controls and MITF siRNAs on MITF and ERBB3 in the WM983B cell line. Representative western blots showing negative siRNA control and MITF siRNAs effect on MITF and ERBB3 in WM983B after 72h. Two different negative siRNA control sequences (NC#1 and NC#2) and three different siRNA sequences against MITF were used (M1-M3). siRNA-induced depletion of MITF was performed in triplicate. Histone H3 was used as loading control.

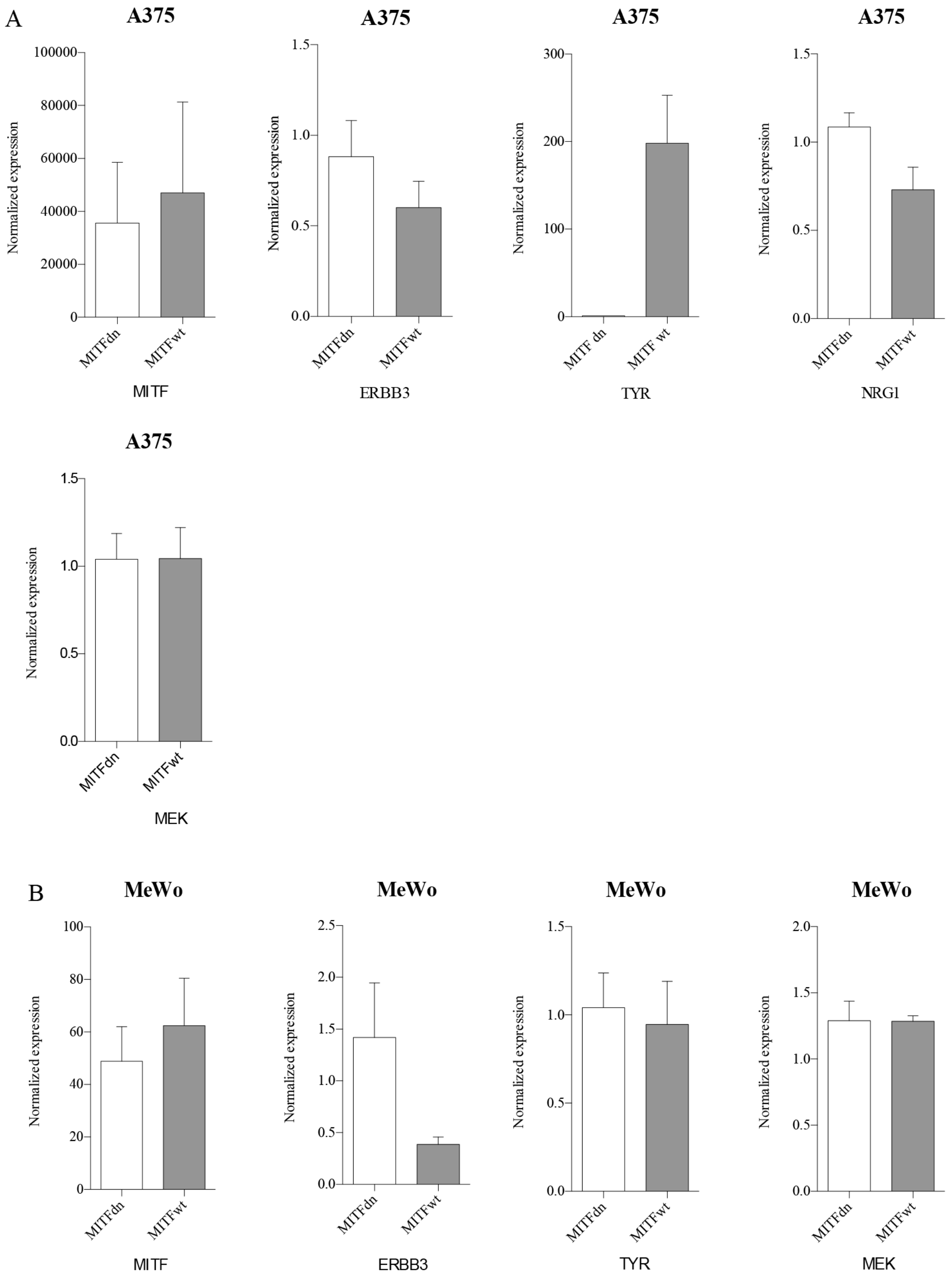

Supplementary Figure S2: MITF overexpression leads to reduction of ERBB3 mRNA levels in A375 and MeWo. HA-tagged MITF wild type (3xHA-tagged MITFvar4) and its nonfunctional control HA-tagged MITF dominant negative (3xHA-tagged MITFdnR215del) was overexpressed in A375 **A.** and MeWo cells **B.** by mRNA transfection. The expression of MITF, ERBB3, TYR and MEK were assessed by qRT-PCR 24hr post mRNA transfection in A375 (A) and MeWo (B) cells. In addition the expression of NRG1-beta was also assessed in A375. Bars represent mean ± SD of three separate experiments.

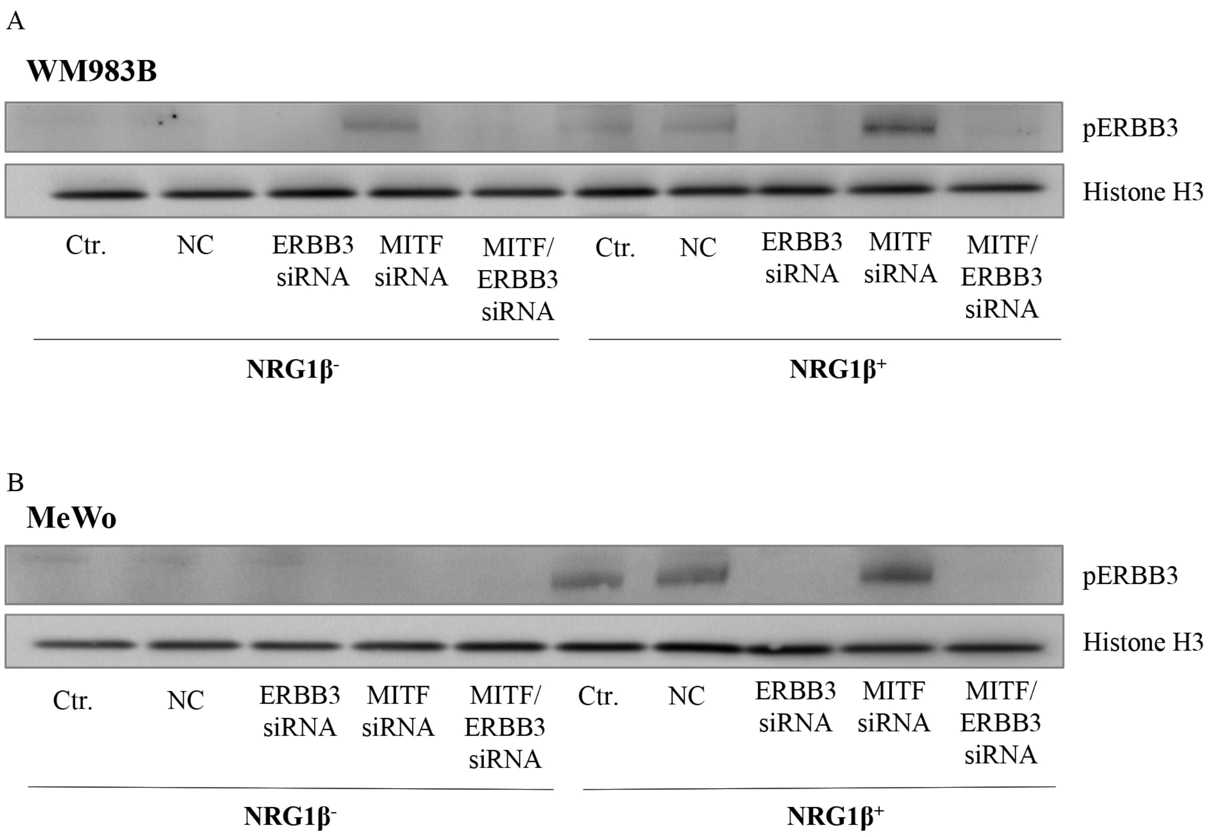

Supplementary Figure S3: The effect of NRG1-beta ligand upon pERBB3 levels. Representative western blots showing pERBB3 levels after various siRNA treatments against ERBB3, MITF, and the ERBB3/MITF combination, either without NRG1-beta ligand or with 10ng/ml NRG1-beta ligand for 15min prior to harvesting.

Supplementary Table S1: Characterization of the cell panel used in the study

Cell line	Stage	BRAF	H/N-RAS	NF1	MITF	ERBB3	SOX10	FOXD3
**Hermes 4C**	**Melanocyte**	WT	WT	WT	3	1	3	3
**Hermes 3c**	**Melanocyte**	WT	WT	WT	3	1	3	2
**WM35**	**Primary**	V600E het	WT	WT	3	2	3	1
**WM793B**	**Primary**	V600E het	WT	WT	0	2	3	1
**WM115**	**Primary**	V600E het	WT	WT	2	3	3	3
**WM1341B**	**Primary**	V600E het	WT	WT	3	2	3	1
**WM1366**	**Primary**	WT	61L	WT	0	0	0	1
**WM983B**	**Metastatic**	V600E het	WT	WT	2	3	3	3
**WM45.1**	**Metastatic**	V600E het	WT	WT	3	2	3	1
**WM239**	**Metastatic**	V600E het	WT	WT	2	2	2	3
**WM266.4**	**Metastatic**	V600E het	WT	WT	2	2	3	3
**WM852**	**Metastatic**	WT	61R	WT	0	0	0	1
**WM1382**	**Metastatic**	WT	WT	WT	2	2	3	3
**LOXIMVI**	**Metastatic**	V600D het	WT	WT	0	0	0	0
**SKMEL28**	**Metastatic**	V600E hom	WT	WT	3	2	3	2
**MeWo**	**Metastatic**	WT	WT	Q1336	2	2	3	1
**A375**	**Metastatic**	V600E hom	WT	WT	1	1	1	1
**WM9**	**Metastatic**	V600E het	WT	WT	0	2	3	2

The table shows cell lines used in the study and their respective disease stage, mutational status (BRAF, RAS, NF1) and relative amount of SOX10/MITF/FOXD3 and ERBB3 mRNA expression levels (3>2>1>0). Strongest inverse association between MITF and ERBB3 expression are marked with black boxes, while medium is marked with grey boxes
